# New Way of Direct Nitrogen Atom Phenylation in Quinoline Derivatives

**DOI:** 10.5402/2012/526867

**Published:** 2012-07-03

**Authors:** Nadezhda E. Shchepina, Viktor V. Avrorin, Gennady A. Badun, Scott B. Lewis, Sergey N. Shurov

**Affiliations:** ^1^Laboratory of Radiochemistry, Natural Sciences Institute, Perm State University, Perm 614990, Russia; ^2^Chemistry Department, Saint Petersburg State University, 26 Universitetsky Pr., Petrodvorets, Saint Petersburg 198504, Russia; ^3^Radiochemistry Department, M. V. Lomonosov Moscow State University, Leninskie Gory, Moscow 119992, Russia; ^4^Department of Chemistry, James Madison University, Harrisonburg, VA 22807, USA; ^5^Department of Organic Chemistry, Perm State University, Perm 614990, Russia

## Abstract

Comparison of ion-molecular reactions of free-phenyl cations generated by tritium **β**-decay with 2-methyl- and 2-phenylquinolines has been investigated. The reaction of direct nitrogen atom phenylation with the help of nucleogenic phenyl cations has been fulfilled for the first time and a new one-step synthesis of tritium-labeled *N*-phenyl-2-phenylquinolinium salt—lipophilic radioactive biological marker has been elaborated.

## 1. Introduction

Among wide variety of heterocyclic compounds, special status undoubtedly belongs to six-ring nitrogen containing heterocycles-pyridine derivatives [1–3]. Ability of nitrogen atom to form lipophilic quaternary onium compounds, the so-called pyridinium salts, together with alterations in its chemical behavior brings new unique possibilities of biological and medicinal applications of these derivatives [4–8]. It was found that in some cases quinolinium compounds reveal essential increase in biologically active properties [9, 10]. Additional introduction of electron-donor methyl group in quinolinium derivatives causes electronic and steric changes in heterocyclic molecule and therefore variation of biological properties [11, 12].

As a result of the manifold aspects of quinolinium compounds' biological application, considerable importance arises in elaboration of new untraditional ways for synthesis of such derivatives together with preparation of radioactive markers containing quinolinium fragment for the detail investigations of chemical and biological processes. It is well-known that classical organic syntheses could not provide the realization of direct nitrogen atom phenylation in pyridine and quinoline derivatives [13, 14]. Previously, we have been shown that application of free nucleogenic phenyl cations generated by tritium *β*-decay within tritium-labeled benzene allows unknown direct nitrogen atom phenylation with the formation of *N-*pyridinium and quinolinium salts [15–17].

In the given work we present comparative investigations of ion-molecular reactions of nucleogenic phenyl cations with aliphatic and aromatic-substituted quinolones.

## 2. Results and Discussions

**Scheme 1:**
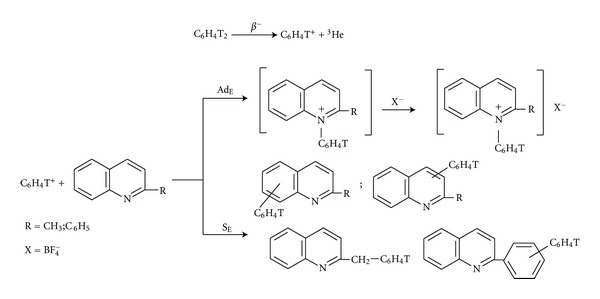
Proposed ion-molecular interactions of nucleogenic phenyl cations with 2-methyl- and 2-phenylquinolines.

Scheme of the investigated ion-molecular interactions might be presented in the following way (Scheme 1).

Nucleogenic phenyl cations were generated by tritium *β*-decay in ditritium benzene. Double labeling gives simultaneously an opportunity to investigate the pathways of proceed reactions with the radioactive label and simple one-step synthesis of tritium-labeled compounds as biological markers. The formed phenylium ions are strong nucleophiles which attack *n-*electrons along with *σ*- and *π-*bonds. Interactions of phenyl cations with the investigated *R*-quinolines are going through electrophilic addition reaction on unshared electron pair of nitrogen atom (quinolinium salts formation) and also through electrophilic substitution reaction on benzene and heterocyclic rings of quinoline (different phenyl substituted quinolines formation). Besides this, the additional ways of interactions might be substitution on methyl group with appearance of benzyl derivatives (in the case of 2-methylquinoline) and substitution on phenyl group in the case of 2-phenylquinoline.

The ion-molecular reactions of *R*-quinolines with the tritium-labeled benzene were carried out in sealed glass ampoules by standard methodic of nuclear-chemical synthesis [15]. The ampoules contained a large excess of a stabilizing salt (KBF_4_). Ditritium-labeled benzene used for this study was synthesized from *p*-dibromobenzene by catalytic exchange with molecular tritium [18]. After an appropriate accumulation time for the determination of products by radioactivity (approximately one month) the reaction mixtures were subjected to TLC analysis. Radiochromatography of tritium-labeled compounds and comparison with the reference compounds (unlabeled *N-*phenylquinaldinium tetrafluoroborate prepared by classical method [19] was used as a reference compound in both cases) allowed determination of the yields of the products.

**Figure 1:**
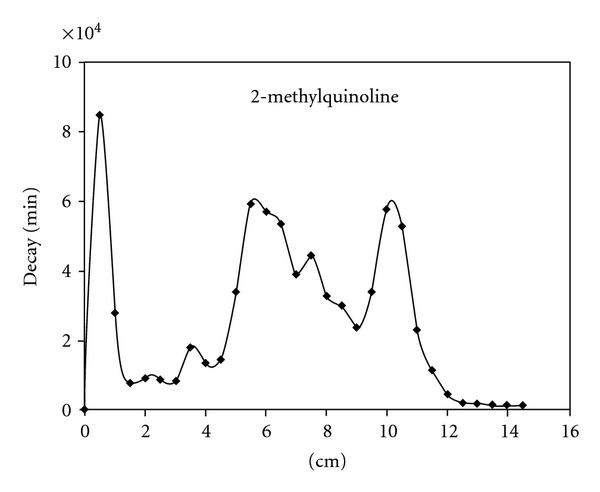
Radiochromatogram of products in the case of C_6_H_4_T_2_—2-methylquinoline—KBF_4_.

**Figure 2:**
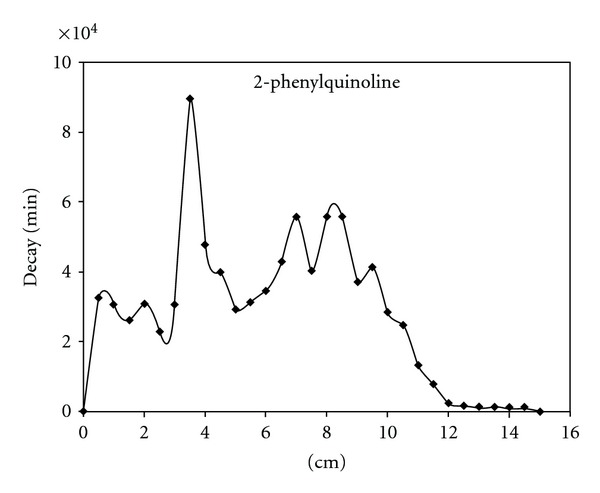
Radiochromatogram of products in the case of C_6_H_4_T_2_—2-phenylquinoline—KBF_4_.

A typical radiochromatogram of the obtained tritiated products from the ion-molecular reactions of tritiated benzene with 2-methyl- and 2-phenylquinolines is presented on Figures 1, 2.

Tritium *β*-radioactivity has been measured by liquid scintillation counter. Relative yields of ion-molecular reactions products were determined as a ratio of the radioactivity of an individual compound towards the sum of all tritium labeled products. Since a wide spectrum of different hardly available labeled substitution-reaction products is formed in both ion-molecular reactions identification and comparison has been done only for the main product of direct nitrogen atom phenylation—*N-*phenylquinolinium salts. Radiochemical yields of quinolinium derivatives are presented in the Table 1.

Methyl substituent into position 2 (2-methylquinoline) slightly decreases the yield of onium derivative (Table 1) due to the steric hindrances of nitrogen atom. Appearance of spatially large phenyl group into *α*-position (2-phenylquinoline) essentially shielding nitrogen atom in heterocyclic ring causes harp decrease in quaternary salt yield (11%, Table 1). At the same time electron-donor substituents presented in heterocyclic ring dramatically shift interactions of nucleogenic phenyl cations which are in singlet form towards electrophilic substitution reaction. This fact becomes certainly evident from the radiochromatograms of the tritiated products (existence of large amount of peaks on the right side of the chromatograms).

## 3. Conclusion

In spite of relatively small yield of onium derivative in the case of 2-phenylquinoline, it is worth to mention that reaction of direct nitrogen atom phenylation with the help of free-phenyl cations generated by tritium *β*-decay has been fulfilled for the first time and a new one-step synthesis of tritium-labeled *N*-phenyl-2-phenylquinolinium salt—lipophilic radioactive biological marker has been elaborated.

## 4. Experimental Section

### 4.1. General Experimental for Nuclear-Chemical Synthesis

All ion-molecular reactions were carried out in sealed glass ampoules (~0.5 cm^3^) containing the source of nucleogenic phenyl cations (ditritium-labeled benzene), the investigated nucleophiles (2-methyl-, 2-phenylquinolines), and an inorganic salt to serve as a stabilizing anion (KBF_4_). In the case of solid 2-phenylquinoline it was previously dissolved in ether or benzene and a solution added to the ampoule with the salt present. The solvent was removed under reduced pressure to coat the salt and finally, ditritiated benzene was added. The molar ratio for ditritium-labeled benzene and substrate was 1 : 1000 (1 *μ*L of hexane solution of tritium double-labeled benzene 1 Ci/cm^3^ and 7.4 *μ*L of 2-methylquinoline or 11.3 mg of 2-phenylquinoline). The ampoules were sealed cold and kept at 0–5°C during accumulation time (about 1 month). After this period, the radioactivity of the obtained products is enough for the detection. The ampoules were opened, unreacted benzene was removed, solvent with unlabeled reference compound added (acetone), and the mixture subjected to TLC analysis on glass plates (*reverse phase C18 silica, fluorescent indicator, *CH_3_CN). Radioactivity of the sorbent layer was measured using a scintillation spectrometer RackBeta 1215 (LKB Wallac, Finland).

### 4.2. Synthetic Preparation of *p*-Ditritium benzene

The reaction of catalytic substitution of halogen atoms by tritium in a molecule of *p-*dibromobenzene serves as a basis for synthesis of tritium double-labeled benzene: from 3.4 mg (0.014 mmol) of dibromobenzene, 5.0 *μ*L (0.020 mmol) tributylamine diluted in 200.0 *μ*L of hexane (addition of tributylamine is necessary for binding of the formed hydrogen bromide), and 3.3 Ci (0.054 mmol) of gaseous tritium by hydrogenation at room temperature on 5% Pd/BaSO_4_ catalyst during 1 h, the solution of tritium double-labeled benzene has been obtained. The chemical purity was not less than 99%. The volume specific activity of the synthesized benzene in hexane solution was 1 Ci/cm^3^.

## Figures and Tables

**Table 1:** Radiochemical yields of *N-*phenylquinolinium derivatives.

Substrate	Yield, % (Ad_E_)
Quinoline	21 ± 3 [16]
2-Methylquinoline	18 ± 2 [16]
2-Phenylquinoline	11 ± 2

## References

[1] Pozharskii AF, Soldatenkov AT, Katritzky AR (1997). *Heterocycles in Life and Society*.

[2] Lukevits E (1995). Pyridine derivatives in the drug arsenal (150 years of pyridine chemistry). *Chemistry of Heterocyclic Compounds*.

[3] Joule JA, Mills K (2000). *Heterocyclic Chemistry*.

[4] Laufer R, Báthori M, Csermely T (2007). TLC determination of hydrophilicity parameter of some pyridinium aldoximes. *Journal of Liquid Chromatography & Related Technologies*.

[5] Dindo D, Dahm F, Szulc Z (2006). Cationic long-chain ceramide LCL-30 induces cell death by mitochondrial targeting in SW403 cells. *Molecular Cancer Therapeutics*.

[6] Bharate SB, Thompson CM (2010). Antimicrobial, antimalarial and antileishmanial activities of mono- and bisquaternary pyridinium compounds. *Chemical Biology and Drug Design*.

[7] Ghosh K, Sarkar T, Chattopadhyay AP (2010). Anthracene appended pyridinium amide-urea conjugate in selective fluorometric sensing of L-N-acetylvaline salt. *Beilstein Journal of Organic Chemistry*.

[8] Kesharwani MK, Ganguly B, Das A, Bandyopadhyay T (2010). Differential binding of bispyridinium oxime drugs with acetylcholinesterase. *Acta Pharmacologica Sinica*.

[9] Stojan J, Marcel V, Fournier D (1999). Inhibition of Drosophila acetylcholinesterase by 7-(methylethoxyphosphinyloxy)1-methyl-quinolinium iodide. *Chemico-Biological Interactions*.

[10] Joaquin CR, Galanakis D, Piergentili A (2000). Synthesis, molecular modeling, and pharmacological testing of bis- quinolinium cyclophanes: potent, non-peptidic blockers of the apamin- sensitive Ca^2+^-activated K^+^ channel. *Journal of Medicinal Chemistry*.

[11] Gutsulyak BM (1972). Biological activity of quinolinium salts. *Russian Chemical Reviews*.

[12] Abd El-Aal RM, Younis M (2004). Synthesis and antimicrobial activity of meso-substituted polymethine cyanine dyes. *Bioorganic Chemistry*.

[13] Pausacker KH (1958). Arylation of aromatic compounds. VI. Benzoyl peroxide with pyridine and quinoline. *Australian Journal of Chemistry*.

[14] Barnes RA, Brody F, Ruby PR (1960). *Pyridine and Its Derivatives*.

[15] Shchepina NE, Avrorin VV, Badun GA, Lewis SB, Fedoseev VM, Ukhanov SE (2009). The reaction of direct phenylation by nucleogenic cations as a method of synthesis of unknown or complicated tritium labeled compounds. *Moscow University Chemistry Bulletin*.

[16] Shchepina NE, Avrorin VV, Badun GA (2009). Preparation of N-phenyl-substituted quinolinium derivatives labeled with tritium by chemonuclear synthesis. *Chemistry of Heterocyclic Compounds*.

[17] Shchepina N, Avrorin V, Badun G, Fedoseev V, Lewis S (2010). New method for the synthesis of difficultly available sterically hindered tritium-labeled pyridinium derivatives. *Chemistry of Heterocyclic Compounds*.

[18] Shchepina NE, Avrorin VV, Badun GA, Fedoseev VM, Ukhanov SE, Lewis SB (2007). Nuclear-chemical synthesis of tritium-labeled phenyl-substituted picoline derivatives. *Radiochemistry*.

[19] Sidorchuk II, Stadniichuk RF, Tishchenko EI, Bordyakovskaya LT (1978). Antimicrobial activity of quaternary quinolinium salts. *Pharmaceutical Chemistry Journal*.

